# Withanolide-Type Steroids from *Withania aristata* as Potential Anti-Leukemic Agents

**DOI:** 10.3390/molecules25235744

**Published:** 2020-12-05

**Authors:** Laila M. Moujir, Gabriel G. Llanos, Liliana Araujo, Angel Amesty, Isabel L. Bazzocchi, Ignacio A. Jiménez

**Affiliations:** 1 Department of Biochemistry, Microbiology, Cell Biology and Genetic, Faculty of Pharmacy, Universidad de La Laguna, Avenida Astrofisico Francisco Sánchez s/n, 38206 La Laguna, Spain; lmoujir@ull.edu.es (L.M.M.); lili_c17@hotmail.com (L.A.); 2 Institute of Bio-Orgánica Antonio González and Organic Chemistry Department, Universidad de La Laguna, Avenida Astrofísico Francisco Sánchez 2, 38206 La Laguna, Spain; ggllanos@gmail.com (G.G.L.); angelamesty@yahoo.es (A.A.); ilopez@ull.edu.es (I.L.B.); 3 Clinical Laboratory Career, Faculty of Health Sciences, Universidad Nacional de Chimborazo, Avenida Antonio José de Sucre, Riobamba 060150, Ecuador

**Keywords:** *Withania aristata*, withasteroids, anti-leukemia activity, structure–activity relationship, drug-likeness prediction

## Abstract

Leukemia is a blood or bone marrow cancer with increasing incidence in developed regions of the world. Currently, there is an ongoing need for novel and safe anti-leukemic agents, as no fully effective chemotherapy is available to treat this life-threatening disease. Herein, are reported the isolation, structural elucidation, and anti-leukemic evaluation of twenty-nine withanolide-type steroids (**1**–**29**) from *Withania aristata*. Among them, the new isolated withanolides, withaperoxidins A–D (**1**–**4**) have an unusual six-membered cyclic peroxide moiety on the withasteroid skeleton as a structural novelty. Their structures have been elucidated by means of spectroscopic analyses, including 2D NMR experiments. In addition, extensive structure–activity relationships and in silico ADME studies were employed to understand the pharmacophore and pharmacokinetic properties of this series of withasteroids. Compounds **15**, **16**, and **22** together with withaferin A (**14**) were identified as having improved antiproliferative effect (IC_50_ ranging from 0.2 to 0.7 μM) on human leukemia HL-60 cell lines compared with the reference drug, etoposide. This cytotoxic potency was also coupled with good selectivity index (SI 33.0–9.2) on non-tumoral Vero cell line and in silico drug likeness. These findings revealed that these natural withasteroids are potential candidates as chemotherapeutic agents in the treatment of leukemia.

## 1. Introduction

Leukemia is a blood or bone marrow cancer characterized by an uncontrolled hematopoiesis process [1]. Acute myeloid leukemia (AML) is a genetically heterogeneous disease that has a poor prognosis and is the most common leukemia in adults. Its incidence has increased dramatically in highly developed regions of the world. AML involves the abnormal proliferation and differentiation of a clonal population of the myeloid stem as a consequence of mutations in the genes implicated in these processes [2] and epigenetic changes [3]. In acute promyelocytic leukemia (APL), the formation of chimeric proteins such as RUNX1-RUNX1T1 and PML-RARA alter the normal maturation process of myeloid precursor cells [4]. Present-day standard intervention for leukemia consists of chemotherapy and stem cell transplantation [5]. Current chemotherapeutic drugs cause a range of side effects and health complications. Moreover, at a time when specific prevention efforts targeting these malignancies are non-existent [6], there is an ongoing need for novel and safe anti-leukemic agents.

Natural products represent a rich source of drug leads, and they have contributed greatly to anticancer drugs discovery and development [7,8]. In this regard, withanolides, naturally occurring C-28-steroidal lactones on an ergostane framework [9], have attracted considerable attention due to their diverse pharmacological properties [10], including anti-inflammatory, immunomodulatory, neurological disorders, and antitumor potential. Withaferin A (WA), the prototype of natural products with withanolide-type skeleton, was investigated for the first time in 1967 as an anticancer agent [11], and since then, a growing list of reports on WA’s broad spectrum antitumor properties [12] and multifunctional molecular mechanism of action [13] have been disclosed. WA molecular biochemical targets against leukemia [14] have also been described. Thus, WA induces cell cycle arrest at G2/M phase [15]) and enhances oxidative stress triggering apoptotic cell death of human myeloid leukemia HL-60 cells [14]. WA induces apoptosis by activation of p38MAPk signaling cascade [16], through downregulation of Akt phosphorylation [17], and by acting as an agonist of liver X receptor a (LXR-α) [18] in leukemia cells.

In the search for new candidates for cancer treatment, we previously identified withanolide-type steroids from *Withania aristata* (Ait.) Pauq. (Solanaceae), an endemic medicinal plant from the Canary Islands [19], and designed a library of withaferin A-analogues as potent inducers in cancer cells [20,21,22,23]. Therefore, encouraged by previous works [14,15,16,17,18], the current study reports the isolation, structure elucidation and anti-leukemic evaluation of four new (**1**–**4**) and twenty-five known (**5**–**29**) withanolides isolated from the acetone extract of *W. aristata*. Their structures were elucidated by detailed spectroscopic analyses, including 2D NMR experiments (COSY, HSQC, HMBC, and ROESY), and comparison with reported data of related compounds. The compounds were tested on HL-60 human cancer cell line, and normal Vero cells searching for selectivity. The structure–activity relationship was analysed, and in silico ADME studies were employed to understand the pharmacokinetic profile of this series of withanolides.

## 2. Results

### 2.1. Chemistry

**Figure 1:**
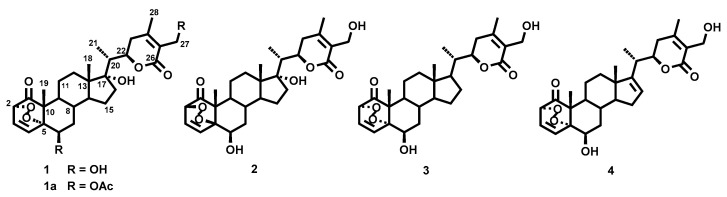
Chemical structures of withaperoxidins A–D (**1**–**4**) isolated from *Withania aristata*.

**Figure 2:**
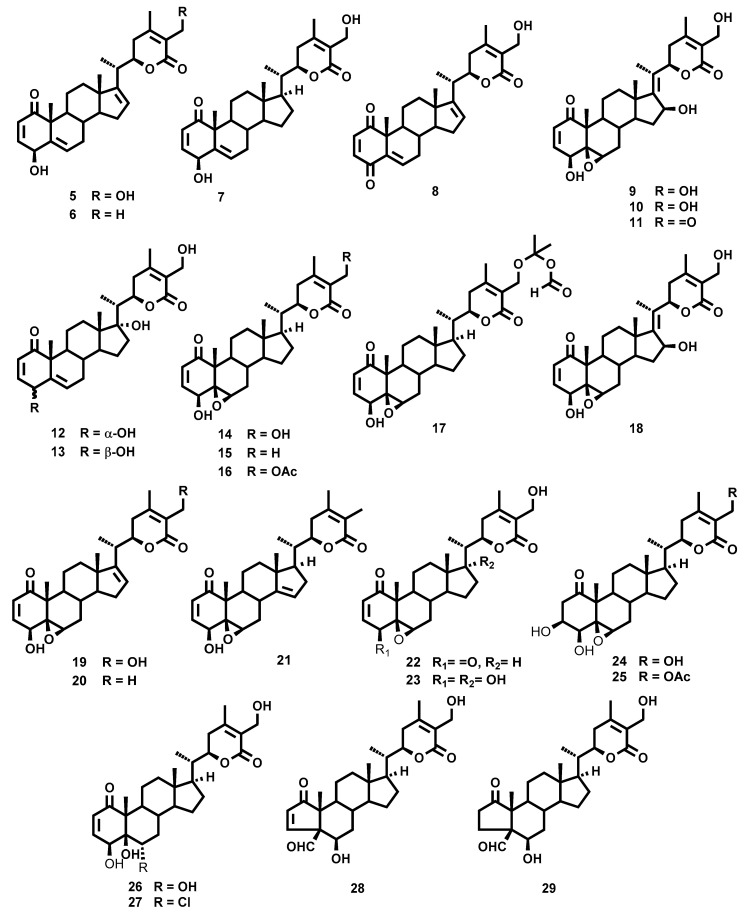
Chemical structures of withanolides **5**–**29** isolated from *Withania aristata*.

The air-dried leaves of *W. aristata* were powdered and extracted with acetone in a Soxhlet apparatus. The crude extract was subjected to multiple chromatographic steps on silica gel and Sephadex LH-20, including vacuum liquid chromatography, medium pressure liquid chromatography as well as preparative TLC and high performance TLC, to yield four new withanolides-type steroids (**1**–**4**) with an unusual cyclic peroxide moiety, along with the known withanolides **5**–**29** (Figure 1 and Figure 2). The structural elucidation was performed as follows.

Compound **1** was isolated as a colorless lacquer with a positive optical rotation [α]^20^_D_ + 20.4. The molecular formula was established as C_28_H_38_O_8_ on the basis of the sodiated molecular [M + Na]^+^ ion peak at *m*/*z* 525.2454 in its HRESIMS, suggesting ten degrees of unsaturation in the molecule. The UV spectrum exhibited a strong absorption at 216 nm, indicating the presence of an α,β-unsaturated carbonyl system, whereas the IR absorption bands revealed the presence of hydroxyl (3740 cm^−1^), carbonyl (1700 cm^−1^), and epoxide (1258 cm^−1^) groups. Its ^1^H-NMR spectrum (Table 1) showed signals for three methyl singlets at δ_H_ 0.88, 1.31, and 2.05, and a methyl doublet at δ_H_ 1.06 (3H, d, *J* = 7.0 Hz) as the most downshift signals. In addition, three oxymethine protons [δ_H_ 4.53 (1H, d, *J* = 6.2 Hz), 4.08 (1H, s), and 4.69 (1H, t, *J =* 8.2 Hz)], two oxymethylene protons [δ_H_ 4.37, 4.42 (2H, d_AB_, *J* = 12.6 Hz)], and two vicinal vinyl protons [δ_H_ 6.69 (1H, dd, *J* = 6.2, 8.2 Hz), and 7.04 (1H, d, *J* = 8.2)], as the most upshift signals were observed. The ^1^H-NMR spectrum in combination with a ^1^H-^1^H COSY experiment allowed the identification of spin systems: H-2/H-3/H-4 in ring A, H-6/H_2_-7 in ring B, and H-20/H_3_-21, H-20/H-22, and H-22/H_2_-23 in the side chain.

In accordance with the molecular formula, the ^13^C-NMR spectrum of **1** (Table 2) exhibited 28 carbon resonances, which were further classified as four methyls, four methylenes, ten methines, and ten quaternary carbons by an HSQC experiment. The downfield region of the ^13^C-NMR spectrum displayed signals for an oxygenated methylene at δ_C_ 57.4, three oxygenated methines (δ_C_ 67.1, 78.6, and 79.2), two oxygenated tertiary carbons (δ_C_ 84.2 and 84.9), two vinyl methines (δ_C_ 126.5 and 141.4), and two olefinic quaternary carbons (δ_C_ 125.1 and 154.3), together with two carbonyl carbons assigned to a δ-lactone (δ_C_ 167.1) and a ketone (δ_C_ 206.0). The aforementioned data suggest that **1** possesses a common tetracyclic withanolide-type steroid skeleton with a ketone, an α,β-unsaturated-δ-lactone, one disubstituted double bond, two oxygenated quaternary groups, two oxymethine groups, and one oxymethylene group (see Figure S1, Supporting Information).

The regiosubstitution of **1** was determined by an HMBC experiment (see Figure S2, Supporting Information), showing as the most relevant three-bond correlations those between the vinyl proton resonance at δ_H_ 7.04 (H-4) and the signals at δ_C_ 78.6 (C-2), 84.2 (C-5), 67.1 (C-6), and 48.2 (C-10), and correlation of the proton resonance at δ_H_ 1.31 (H_3_-19) and signals at δ_C_ 206.0 (C-1), 84.2 (C-5), 42.1 (C-9), and 48.2 (C-10), locating the ketone at C-1, a double bond at C-3/C-4, and the oxygenated groups at C-2, C-4, C-5, and C-6. Correlations observed from methyl resonances at δ_H_ 1.06 (H_3_-21) and 0.88 (H_3_-18) to the signal at δ_C_ 84.9 (C-17) confirmed the structure of the D ring, whereas the signal at δ_H_ 2.05 (H_3_-28) was linked to the resonance at δ_C_ 32.9 (C-23), 154.3 (C-24), and 125.1 (C-25), establishing the structure of the α,β-unsaturated-δ-lactone on the side chain. All these data accounted for nine out of 10 degrees of unsaturation established by its HRESIMS spectrum, indicating that compound **1** has an additional ring in the withanolide skeleton. The presence of a 1,2-dioxane system is the only option that satisfies this chemical shift requirements, which can be attributed to a six- or four-membered cyclic peroxide ring system at C-2/C-5 or C-5/C-6. This naturally occurring cycloendoperoxide would arise from a [4 + 2] or [2 + 2] cycloaddition reaction between the cyclohexadiene system ∆^2,4^ or double bond ∆^5^ on the withonolide skeleton and in situ formed singlet oxygen [24].

The regiosubstitution of the cyclic peroxide was resolved by chemical transformation. Thus, derivative **1a** was prepared by standard acetylation of compound **1** with acetic anhydride. The main difference in its 1D NMR (see Figure S3, Supporting Information) was the replacement of the hydroxyl groups at C-6 and C-27 in **1** by two acetate groups in **1a**, together with the downfield shift of the signals corresponding to H-6 and H_2_-27. These data suggest that **1a** is the 6,27-diacetyl derivative of **1** and the six-membered cyclic peroxide ring system is located at C-2/C-5. Moreover, the structure of **1a** was verified by 2D NMR spectroscopic analysis in combination with spectrometric studies.

**Figure 3:**
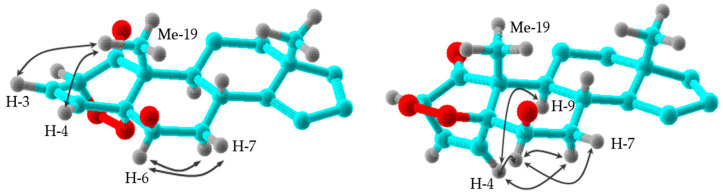
Selected ^1^H-^1^H ROESY correlations of withaperoxidins A (**1**) and B (**2**).

The relative configuration of **1** was established based on the coupling constants, molecular mechanic calculation using the PC Model [25], comparison with reported data of **23** [26], and confirmed by a ROESY experiment. Thus, the relative stereochemistry of the C-2/C-5 cyclic peroxide moiety was established by correlation of the H_3_-19 with H-3 and H-4. Moreover, a cross-peak of H-6α with H-7α and H-7β confirmed the β-axial stereochemistry of the hydroxyl group at C-6. Furthermore, the β-stereochemistry of the hydroxyl group at C-17 was determined by the correlations of H_3_-18 with H-20 and H_3_-21 (Figure 3). Therefore, the structure of **1** was established as 6β,17α,27-trihydroxy- 2α,5α-dioxan-1-oxo-witha-3,24-dien-26,22-olide, named withaperoxidin A.

Withaperoxidin B (**2**) was assigned an identical molecular formula (C_28_H_38_O_8_) to that of **1** by HRESIMS. A study of IR, UV, ^1^H, and ^13^C-NMR (Table 1 and Table 2) and 2D spectra showed it to be a withanolide-type steroid with a carbonyl group (δ_C_ 205.9, C-1), a disubstituted endocyclic double bond [δ_H_ 6.67 (dd, *J* = 6.4, 8.1 Hz, H-3) and 6.58 (d, *J* = 8.1 Hz, H-4); δ_C_ 125.9 (C-3) and 142.5 (C-4)], a primary hydroxyl group [δ_H_ 4.37, 4.41 (d_AB_, *J* = 11.8 Hz, H-27), and δ_C_ 57.5 (C-27)], three oxymethine protons [δ_H_ 4.60 (d, *J* = 6.4 Hz, H-2), 4.15 (s, H-6), and 4.67 (dt, *J* = 2.9, 11.8 Hz, H-22); δ_C_ 80.0 (C-2), 69.1 (C-6), and 79.1 (C-22)], two tertiary hydroxyl groups [δ_C_ 84.6 (C-5) and 85.0 (C-17)], and an α,β-unsaturated δ-lactone moiety [δ_C_ 154.1 (C-24), 125.2 (C-25), and 167.1 (C-26)]. These NMR data show **2** to be similar to **1**. The most significant differences were the downfield shift of H_3_-19 (Δδ 0.30) and those of C-2 (Δδ 1.4) and C-6 (Δδ 2.0), and an upfield shift of H-4 (Δδ 0.46) and C-19 (Δδ 3.4) in the ^1^H and ^13^C-NMR spectra (see Figure S4, Supporting Information). Analysis of 2D spectra (see Figure S5, Supporting Information) indicated that **2** is a stereoisomer of **1** at the 2,5-endoperoxide moiety, which was confirmed by a ROESY experiment. Thus, ROE correlations of H-4/H-6, H-7α, and H-9 defined the β-stereochemistry of the C-2/C-5 cyclic peroxide group (Figure 3). Therefore, the structure of **2** was established as 6β,17α,27-trihydroxy-2β,5β-dioxan-1-oxo-witha-3,24-dien-26,22-olide.

The HRESIMS of withaperoxidin C (**3**) gave a molecular formula C_28_H_38_O_7_, indicating an oxygen atom less than **1**. Its ^1^H and ^13^C-NMR data (Table 1 and Table 2, and Figure S6, Supporting Information) showed that **3** has similar features to those of compound **1**, with the only difference being the replacement of the tertiary hydroxyl group at C-17 (δ_C_ 84.9, s) in **1** by a methine group [(δ_H_ 1.16, m) and (δ_C_ 51.9, d)] in **3**, indicating that **3** is the 17-deoxy analogue of **1**. Analysis of 2D NMR data allowed the complete and unambiguous assignment of chemical shifts, regiosubstitution, and relative configuration of compound **3**. The HMBC experiment showed as the most relevant three-bond correlations those of the proton resonances of H_3_-18 (δ_H_ 0.79) and H_3_-21 (δ_H_ 1.05) with the signal of C-17 (δ_C_ 51.9). Accordingly, the structure of withaperoxidin C (**3**) was defined as 6β,27-dihydroxy-2α,5α-dioxan-1-oxo-witha-3,24-dien-26,22-olide.

Compound **4** showed spectral data resembling those of compound **1**. Its molecular formula (C_28_H_36_O_7_), determined by HR-ESIMS, revealed 18 mass units less than the parent **1**. A comparison of their NMR spectra showed the replacement of the signals assigned to the aliphatic H_2_-16 and the tertiary hydroxyl group at C-17 in compound **1** by resonances for a trisubstituted double bond [δ_H_ 5.55 (1H, s, H-16), δ_C_ 124.4 (CH-16) and 155.3 (C-17)] in **4**. Even so, it should be noted that a set of 2D NMR spectra was performed for **4** with the aim of gaining the full and unequivocal assignment of the ^1^H and ^13^C-NMR resonances (Table 1 and Table 2, and Figure S7, Supporting Information). The regiosubstitution of functional groups on the steroid skeleton was determined by an HMBC experiment and the relative stereochemistry by a ROESY experiment. This spectral evidence established the structure of withaperoxidin D (**4**) as 6β,27-dihydroxy-2α,5α-dioxan-1-oxo-witha-3,16,24-trien-26,22-olide.

The structure of the twenty-five known compounds were identified by comparison with their observed and reported NMR data as follows: 4β,27-dihydroxy-1-oxo-witha-2,5,16,24-tetraenolide (**5**) [20], 4β-hydroxy-1-oxo-witha-2,5,16,24-tetraenolide (**6**) [20], 4β,27-dihydroxy-1-oxo-witha-2,5,24-trien olide (**7**) [27], 27-hydroxy-1,4-dioxo-witha-2,5,16,24-tetraenolide (**8**) [20], 4β,16β,27-trihydroxy-1-oxo-witha-2,5,17(20),24-tetraenolide (**9**) [20], 4β,16β-dihydroxy-1-oxo-witha-2,5,17(20),24-tetraenolide (**10**) [20], 16β,27-dihydroxy-1,4-dioxo-witha-2,5,17(20),24-tetraenolide (**11**) [20], 4α,17α,27-trihydroxy-1-oxo-witha-2,5,24-trienolide (**12**) [20], 4β,17α,27-trihydroxy-1-oxo-witha-2,5,24-trienolide (**13**) [28], withaferin A (**14**) [29], 5β,6β-epoxy-4β-hydroxy-1-oxo-witha-2,24-dienolide (**15**) [30], 27-acetyl-withaferin A (**16**) [21], 27-*O*-ispropylformate-withaferin A (**17**) [21], 5β,6β-epoxy-4β,16β,27-trihydroxy-1-oxo-witha-2,17(20),24-trienolide (**18**) [21], witharistatin (**19**) [31], 27-deoxy-16-en-withaferin A (**20**) [32], 5β,6β-epoxy-4β-hydroxy-1-oxo-22*R*-witha-2,14,24-trienolide (**21**) [33], 4-dehydro-withaferin A (**22**) [34], 5β,6β-epoxy-4β,17α,27-trihydroxy-1-oxo-witha-2,24-dienolide (**23**) [26], viscosalactone B (**24**) [35], 27-*O*-acetyl-viscosalactone B (**25**) [21], 2,3-dehydrosomnifericin (**26**) [36], 6α-chloro-5β-hydroxywithaferin A (**27**) [27], 4β-formyl-6β,27-dihydroxy-1-oxo-witha-2,24-dienolide (**28**) [21], and 4β-formyl-6β,27-dihydroxy-1-oxo-witha-24-enolide (**29**) [21].

These endoperoxides were isolated from the acetone extract of *W. aristata*; however, these metabolites were not detected in previous works on the dichloromethane extract from this plant [20,21,22]. This may be due not only to their solubility, but also to their stability in the selected solvent. Thus, withaperoxidins remain chemically unaltered in acetone but not in dichloromethane. This supports that the solvent system is one of the most important steps in extract preparation for a phytochemical. Endoperoxides have received widespread attention due to their ubiquitous structures, very sporadic occurrence, and promising biological properties. So far, more than 1000 peroxides have been structurally characterized from natural sources, while only about two hundred endoperoxides have been isolated from plants [37]. To date, five highly oxygenated withanolides with an endoperoxide bridge have been characterized from *Physalis alkekengi* [38,39] and *Jaborosa odonelliana* [40]. However, to our knowledge, this is the first report of withasteroid-endoperoxides from *Withania* genus, suggesting that the enzymatic system in *W. aristata* is quite different from other species of this genus, which may have some biogenetic implications.

### 2.2. Antiproliferative Activity

The twenty-nine compounds (**1**–**29**) isolated from the acetone extract of *W. aristata* were assayed for their in vitro antiproliferative activity against the human promyelocytic leukemia cell line HL-60, and the Vero (African green monkey kidney) non-tumoral cell line used for selectivity purposes. Among the isolated withasteroids, except for withaferin A (**14**), previously investigated on various leukemia cell lines [14,15,16,17,18], there is only one earlier report from the 80 s on the anti-leukemic activity of the known isolated compound **22** [41]. Thus, we included both compounds because the cell lines and procedures were different from those used herein.

The results obtained indicate (Table 3) that eight compounds (**14**–**16**, **19**, **21**, **22**, **25,** and **27**) exhibit higher antiproliferative activity (IC_50_ values ranging from 0.2 to 1.5 μM) against the tested tumor cell line than the reference drug, etoposide (2.4 μM). Among them, WA, followed by withasteroids **16**, **15**, and **19** showed IC_50_ values ≤ 0.5 μM, meaning 12.0 to 4.8-fold more potent than the reference drug. Furthermore, ten of this series of compounds (**3**–**6**, **8**, **12**, **17**, **18**, **20,** and **23**) also displayed good cytotoxicity with IC_50_ values ranging from 3.3 to 8.2 μM. In addition, some degree of selective cytotoxicity was observed when comparing HL-60 cancer cells with the non-tumorigenic Vero cell line (Table 3). Thus, it is noteworthy that withasteroids **14**–**16** and **22**, among the most active compounds, exhibited an excellent selectivity index (SI) with values of 33.0, 9.2, 14.8, and 24.1, respectively, also higher than that of the positive control (SI 8.2).

We assume that a selectivity index (SI) value higher than two indicates good selectivity for inducing cytotoxicity in tumor cell lines as compared to those in noncancerous cells, according to Suffness [42]. Thus, among the tested compounds, 21 of them showed selectivity to some extent (SI > 2) in the non-tumoral Vero cell line with respect to the HL-60 cell line. The overall results of the biological assays identified withasteroids **15**, **16**, and **22**, in addition to the well-known anti-cancer lead compound, withaferin A (**14**), as having significantly improved activity profiles compared with the reference drug, etoposide. Moreover, their increased selectivity index makes these withasteroids suitable for further studies. The results have expanded the structural diversity of withanolides and opened up new possibilities to identify new lead compounds based on the WA skeleton.

### 2.3. Structure–Activity Relationship Analysis

The influence of the substitution pattern of the withanolide-type skeleton and its connection with anti-leukemic activity was examined, revealing the following trends of this series of natural withanolides: (a) In ring A, a double bond at the C-2 position was highly favorable (**14,** IC_50_ 0.2 μM versus **24**, IC_50_ > 40 μM). Notably, the cytotoxic effect of the 27-acetylwithanolide **25** (IC_50_ 1.5 μM) was 26-fold more potent than its corresponding hydroxyl derivative **24**, suggesting that the enhancement of potency could be correlated to the lipophilicity of the molecule. No straightforward conclusion can be drawn from the presence of hydroxyl group or ketone group at C-4, given that their existence displayed increases (**5** versus **8**) and decreases (**15** versus **22**) in antiproliferative activity. The stereochemistry of the hydroxyl group at C-4 has a moderate effect on activity (**12** resulted 2-fold more potent than the β-disposition (**13**) in contrast to previous findings [43]. (b) Analysis of the role of the functional group at B ring showed that withanolides with an epoxy group at C-5/C-6 displayed more potency than those with a double bond moiety (**14**, **18**, **19**, **20**, and **23** versus **7**, **9**, **5**, **6,** and **13**, respectively). By contrast, dihydroxylation at C-5/C-6 has a detrimental effect on the activity (**14** vs. **26**). In agreement with previous results [22], the cytotoxic effect of the 6-chlorowithanolide **27** was 12-fold more potent than its corresponding hydroxyl derivative **26**, indicating that the enhancement of potency appears to be correlated to the lipophilicity of substituents at C-6. (c) In general, substituents on the five member D ring play a moderate role according to previous results [20]. Thus, a detrimental effect was observed in Δ^16^-withanolides over the corresponding congeners (**4**, **7**, **19,** and **20** vs. **3**, **5**, **14**, and **15**, respectively) and those with a hydroxyl group at C-17 (**3** vs. **1** and **14** vs. **23**), whereas withasteroids with a hydroxyl group at C-16 and double bond at C-17/C-20 exhibited a dramatic decrease in cell proliferation (**9**–**11**). (d) Regarding the lactone ring, results indicated that the substituent at C-27 on the core skeleton plays a notable role on activity. Compounds with a hydroxyl-methylene group (**14** and **19**) showed better in vitro activity on the HL-60 cell line than those with a methyl group (**15** and **20**), suggesting that it may be involved in H-bonding with the target. Moreover, a ketal group at C-27 resulted in a significant drop of potency (**17** vs. **14**). (e) SAR analysis of withanolides **1**–**4** showed that the replacement of the typical system enone and epoxy at the A and B rings for the unusual cycloendoperoxide moiety was unfavorable, leading to a broad range of activities (IC_50_ values ranging from 3.3 to 26.7 μM), depending largely on the substituent at C-17 (**1** and **2** vs. **3** and **4**), suggesting the enhancement of potency appeared to be correlated to the lipophilicity of the substituents on the D-ring and, to a lesser extent, on the stereochemistry of the endoperoxide (**1** vs. **2**). Furthermore, the structure–selectivity relationship analysis is in agreement with the anti-leukemic activity. Thus, the double bond at C-2 (**14** vs. **24**), the C-5/C-6 epoxy group (**14**, **18**, **19,** and **23** vs. **7**, **9**, **5,** and **13**, respectively) and the hydroxyl-methylene group at C-27 (**14** vs. **15** and **16**) play a favorable role on the selectivity index, increasing from 2.3 to 33.0-fold. However, substituents on D-ring have a detrimental effect on the selectivity (**14** vs. **19**, **14** vs. **23,** and **15** vs. **20**), decreasing between 4.2 and 8.7-fold.

**Figure 4:**
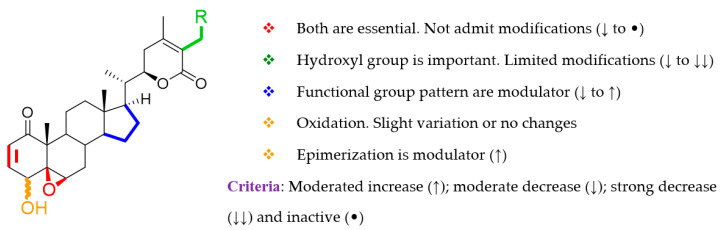
Overview of the structural requirements of isolated withanolides based on structure–anti-leukemic activity relationship analysis.

In summary, simple modifications of both essential groups, a double bond at C-2/C-3, and an epoxy group at C-5/C-6, produced a clear decrease in activity, as occurs in endoperoxides **1**–**4**, Michael adduct **24**, and withanolides **28**–**29** with an A-ring rearrangement. These results agree with previous studies [21,22,43], which define these functions as essential structural requirements in the withanolide skeleton for optimum antiproliferative activity. Moreover, substituents on D and lactone rings open up new challenges to modulate the antileukemic activity (Figure 4).

### 2.4. In Silico ADMET Predictions

In silico prediction of pharmacokinetic properties such as drug absorption, distribution, metabolism, excretion, and toxicity (ADMET) has become an important tool to select new lead/drug candidates and help in anticipating clinical suitability [44]. Although withanolides have been extensively investigated for their anticancer properties, there are only three reports on their ADMET studies [23,45,46]. In order to increase the success rate of compounds reaching further stages of development, the QikProp module of Schrödinger software [47] can be used as a computational method for analyzing the pharmacokinetic descriptors of the compounds (Table 4).

The parameters analyzed provide insights into key aspects such as drug likeness, permeability, solubility, bioavailability, oral absorption, metabolism, etc. A detailed account of these parameters for compounds **1**–**29** is given in Table S1, Supporting Information. The first parameter that is taken into account is #stars. This identifies the number of properties of each compound that fail to remain within the recommended ranges; therefore, a lower number of #stars represents a better drug-like molecule [47]. Chemo-informatics analysis showed that all the compounds complied with this descriptor with excellent values. Thus, taking into consideration the IC_50_ values of the assayed withanolides with higher cytotoxic effect than etoposide (IC_50_ < 2.4 μM on HL-60 cell line), compounds **14**–**16**, **19**, **21**, **22**, **25**, and **27** were selected to analyze their predicted pharmacokinetic properties (Table 4). Predicted values for all compounds fit the lipophilicity parameter (QP logPo/w), an important physicochemical property requirement for a potential drug, as it plays a crucial role in absorption, bioavailability, hydrophobic drug-receptor interactions, metabolism, excretion, toxicity, and in vivo pharmacological properties [48]. Distribution depends on various parameters such as the amount of binding of the drug to plasma proteins (log Khsa), the volume of body fluid that is required to dissolve it, and central nervous system permeability (QP log S). In this sense, all compounds had values in accordance with standard values. A descriptor of metabolic reactions (#metab) determines how efficiently a drug is converted to metabolites after being absorbed, distributed, and excreted, and the compounds showed ideal ranges. In this way, the predicted values of selected compounds for properties such as molecular weight, number of hydrogen-bond donor, number of hydrogen-bond acceptor, number of rotatable bonds, solubility in water, surface area, octanol/water partition, gut-blood barrier permeability, human serum albumin binding, and percentage of human oral absorption lie well within the recommended ranges. Therefore, the above data indicate that the most potent compounds, withasteroids **15**, **16**, and **22**, in addition to WA (**14**), exhibited very good drug-likeness, since they obeyed Lipinski’s rule of 5 and did not inflict adverse effects, as well as meeting all the pharmacokinetic criteria. Therefore, they can be considered as candidate leads.

## 3. Materials and Methods

### 3.1. General

Optical rotations were measured on a Perkin Elmer 241 automatic polarimeter (Waltham, MA, USA) in CHCl_3_ at 20 °C, and the [α_D_] are given in 10^−1^ deg cm^2^ g^−1^. UV spectra were obtained on a JASCO V-560 spectrophotometer (JASCO-Europe, Cremelle, Italy), and IR (film) spectra were measured on a Bruker IFS 55 spectrophotometer (Bruker Co. Billerica, MA, USA). NMR experiments were performed on a Bruker Avance 400 spectrometer (Bruker Co. Billerica, MA, USA), and chemical shifts are shown in δ (ppm) with tetramethylsilane (TMS) as internal reference. EIMS and HREIMS were recorded on a Micromass Autospec spectrometer, and ESIMS and HRESIMS (positive mode) were measured on an LCT Premier XE Micromass Electrospray spectrometer (Micromass, Manchester, UK). Purification was performed using silica gel 60 μM for column chromatography (particle size 15–40 and 63–200 μm), POLYGRAM SIL G/UV_254_ used for analytical and preparative TLC, and HPTLC-platten Nano-Sil 20 UV_254_ were purchased from Panreac (Barcelona, Spain). Sephadex LH-20 for exclusion chromatography was obtained from Pharmacia Biotech (Pharmacia, Uppsala, Sweden). The spots in the TLC were visualized by UV light and heating silica gel plates sprayed with H_2_O-H_2_SO_4_-AcOH (1:4:20). All solvents used were analytical grade from Panreac (Barcelona, Spain). Reagents were purchased from Sigma Aldrich (St Louis, MO, USA) and used without further purification.

### 3.2. Biological Assays

#### 3.2.1. Cells

HL-60 (human promyelocytic leukemia cell line) and Vero (African green monkey kidney) cell lines from American Type Culture Collection (ATCC-LGC) were each grown in Dulbecco’s modified Eagle’s medium (DMEM) with 4.5 g/L glucose (Sigma-Aldrich), supplemented with 10% fetal bovine serum (Gibco), 1% of a penicillin–streptomycin mixture (10.000 and 10 mg/mL, respectively), and 200 mM L-glutamine. Cells were maintained at 37 °C in 5% CO_2_ and 98% humidity.

#### 3.2.2. Cell Viability

Viable cells were assessed using the colorimetric MTT [3-(4,5-dimethylthiazol-2-yl)-2,5-diphenyl tetrazolium bromide reduction assay [49]. A cell suspension (1 × 10^4^ /200 μL/well) in log-phase of growth seeded in DMEM, supplemented with 5% fetal bovine serum in microtiter well plates (96 wells, Iwaki, London, UK) were added to compounds predissolved in dimethylsulphoxide (DMSO) at different concentrations. After 48 h of incubation, 20 μL/well of the MTT solution (5 mg/mL in phosphate buffered saline, PBS) were added and plates incubated for 4 h at 37 °C. Subsequently, the medium/MTT solutions were removed carefully by aspiration and replaced with 150 μL/well of DMSO to dissolve the formazan crystals. Absorbance was measured in a microplate reader (Infinite M200, Tecan) at a wavelength of 550 nm. Solvent control cultures were considered 100% and percentage of viable treated cells were plotted against compound concentrations. The 50% cell viability value (IC_50_) was calculated from the curve. Each experiment was performed at least three times in triplicates. Data are given in arithmetic means ± SD. Selectivity ratio was defined as IC_50_ value for the tumoral cell line divided by IC_50_ value for the Vero cell line.

### 3.3. Plant Material

Leaves of *W. aristata* were collected in Icod de los Vinos, Tenerife, Canary Islands (Spain), in May 2005. A voucher specimen (TFC 48.068) is deposited in the Herbarium of the Department of Botany, University of La Laguna, Tenerife, and identified by Leticia Rodríguez-Navarro.

### 3.4. Extraction and Isolation

The air-dried powdered leaves of *W. aristata* (1.8 kg) were exhaustively extracted with acetone in a Soxhlet apparatus, and the solvent was evaporated at reduced pressure. The residue (80 g) was fractioned by vacuum-liquid chromatography on silica gel and eluted with hexanes/EtOAc mixtures of increasing polarity (from 100:0 to 0:100) affording thirty-four fractions, which were combined on the basis of their TLC profiles in six fractions (F1 to F6). Preliminary ^1^H-NMR analysis revealed that fractions F3 to F5 were rich in withanolides and were further investigated. Each of these fractions was subjected to column chromatography over Sephadex LH-20 (hexanes/CHCl_3_/MeOH, 2:1:1) and silica gel (CH_2_Cl_2_/acetone of increasing polarity). Preparative and high performance thin-layer chromatography developed with CH_2_Cl_2_/acetone (8.5:1.5) and CH_2_Cl_2_/EtOAc (8:2) were used to purify the new compounds **1** (3.9 mg), **2** (3.4 mg), **3** (3.2 mg), and **4** (3.1 mg), in addition to the known compounds **5** (2.0 mg), **6** (1.9 mg), **7** (1.0 mg), **8** (1.5 mg), **9** (14.5 mg), **10** (4.0 mg), **11** (2.2 mg), **12** (1.9 mg), **13** (28.0 mg), **14** (1.7 g), **15** (1.9 mg), **16** (10.2 mg), **17** (1.1 mg), **18** (1.4 mg), **19** (45.2 mg), **20** (2.7 mg), **21** (2.1) mg, **22** (1.2 mg), **23** (5.7 mg), **26** (13.2 mg), **27** (9.0 mg), **28** (3.1 mg), and **29** (1.3 mg).

1. 6β,17α,27-Trihydroxy-2α,5α-dioxan-1-oxo-witha-3,24-dien-26,22-olide (withaperoxidin A, 1). Colorless lacquer; [α]_D_^20^ + 20.4 (c 0.3, CHCl_3_); UV λ_max_ 216 nm; IR ν_max_ 3740, 2929, 2862, 1700, 1454, 1399, 1258, 1060, 830 cm^−1^; ^1^H and ^3^C-NMR data (see Table 1 and Table 2, respectively); ESIMS *m*/*z* % 525 [M + Na]^+^ (100); HRESIMS *m*/*z* 525.2454 (calcd for C_28_H_38_O_8_Na, 525.2464).
2. Acetylation of **1**. To a solution of **1** (7.0 mg, 0.12 mmol) in dry dichloromethane (2 mL) was added triethylamine (0.1 mL, 0.8 mmol) and acetic anhydride (0.15 mL, 0.12 mmol). The reaction mixture was stirred for 72 h at room temperature until all starting material was consumed. The mixture was evaporated to dryness. The residue was then purified by preparative TLC (dichloromethane/acetone, 9:1) to give **1a** (4.5 mg, 56%): Colorless lacquer; [α]_D_^20^ + 56.0 (*c* 0.2, CHCl_3_); UV λ_max_ 223 nm; IR ν_max_ 2925, 1697, 1454, 1393, 1255, 1063, 834 cm^−1^; ^1^H-NMR δ 0.88 (3H, s, Me18), 1.05 (3H, d, *J* = 6.9 Hz, Me21), 1.14 (3H, s, Me19), 1.21 (1H, H15), 1.39 (1H, H12), 1.52 (1H, H11), 1.61 (1H, H16), 1.63 (1H, H12), 1.68 (1H, H8), 1.69 (1H, H11), 1.71 (1H, H14), 1.72 (1H, H7α), 1.73 (1H, H16), 1.76 (1H, H15), 1.98 (1H, H9), 2.00 (1H, H7β), 2.09 (3H, s, Me28), 2.36 (1H, m, H20), 2.59 (2H, d, *J* = 7.8 Hz, H23), 4.54 (1H, d, *J* = 6.2 Hz, H2), 4.68 (1H, dt, *J* = 3.0, 8.8 Hz, H22), 4.89, 4.93 (2H, d_AB_, *J* = 11.8 Hz, H27), 5.38 (1H, br s, H6), 6.62 (1H, t, *J* = 8.5 Hz, H3), 7.00 (1H, d, *J* = 8.5 Hz, H4), OAc-27 [2.09 (3H, s)], OAc-6 [2.16 (3H, s)]; ^13^C-NMR δ 9.4 (CH_3_-21), 15.0 (CH_3_-18), 18.0 (CH_3_-19), 20.6 (CH_3_-28), 21.8 (CH_2_-11), 23.6 (CH_2_-15), 29.8 (CH-8), 31.9 (CH_2_-16), 32.0 (CH_2_-12), 33.1 (CH_2_-23), 36.5 (CH_2_-7), 42.6 (CH-20), 43.5 (CH-9), 47.1 (C-10), 48.3 (C-13), 49.1 (CH-14), 58.0 (CH_2_-27), 69.8 (CH-6), 78.7 (CH-2), 79.1 (CH-22), 85.0 (C-5), 85.9 (C-17), 121.3 (C-25), 130.9 (CH-3), 142.9 (CH-4), 158.6 (C-24), 165.5 (C-26), 205.4 (C-1), OAc-6 [20.8 (CH_3_), 169.6 (COO)], OAc-27 [21.0 (CH_3_), 171.0 (COO)]; ESI/MS *m*/*z* % 609 [M + Na]^+^ (100); HRESIMS *m*/*z* 609.2679 (calcd for C_32_H_42_O_10_Na, 609.2676).
3. 6β,17α,27-Trihydroxy-2β,5β-dioxan-1-oxo-witha-3,24-dien-26,22-olide (withaperoxidin B, **2**). Colorless lacquer; [α]_D_^20^ + 31.9 (*c* 0.2, CHCl_3_); UV λ_max_ 214 nm; IR ν_max_ 3430, 2929, 2855, 1689, 1457, 1394, 1013, 754 cm^−1^; ^1^H and ^3^C-NMR data (see Table 1 and Table 2, respectively); ESIMS *m*/*z* % 525 [M + Na]^+^ (100); HRESIMS *m*/*z* 525.2465 (calcd for C_28_H_38_O_8_Na, 525.2464).
4. 6β,27-Dihydroxy-2α,5α-dioxan-1-oxo-witha-3,24-dien-26,22-olide (withaperoxidin C, **3**). Colorless lacquer; [α]_D_^20^ − 3.9 (*c* 0.3, CHCl_3_); UV λ_max_ 217 nm; IR ν_max_ 3444, 2927, 1700, 1458, 1393, 1130, 1024, 754 cm^−1^; ^1^H and ^3^C-NMR data (see Table 1 and Table 2, respectively); ESI/MS *m*/*z* % 509 [M + Na]^+^ (100); HRESIMS *m*/*z* 509.2508 (calcd for C_28_H_38_O_7_Na, 509.2515).
5. 6β,27-Dihydroxy-2α,5α-dioxan-1-oxo-witha-3,16,24-trien-26,22-olide (withaperoxidin D, **4**). Colorless lacquer; [α]_D_^20^ − 4.0 (*c* 0.5, CHCl_3_); UV λ_max_ 214 nm; IR ν_max_ 3410, 2930, 1681, 1461, 1380, 1131, 1078, 755 cm^−1^; ^1^H and ^3^C-NMR data (see Table 1 and Table 2, respectively); ESI/MS *m*/*z* % 507 [M + Na]^+^ (100); HRESIMS *m*/*z* 507.2355 (calcd for C_28_H_36_O_7_Na, 507.2359).

### 3.5. ADME Property Predictions of Withanolides

Prediction of descriptors related to absorption, distribution, metabolism, and excretion (ADME) properties of the compounds were predicted using the QikProp program (QikProp, version 6.3) [47] in Fast mode and based on the method of Jorgensen [50,51]. Preparation of compounds and the 2D-to-3D conversion was performed using LigPrep tool, a module of the Small-Molecule Drug Discovery Suite in Schrödinger software package (version 2020-1), followed by a MacroModel 12.3 Monte Carlo conformational search to locate the lowest energy conformation of each ligand. The program computes pharmacokinetic relevant properties such as octanol/water partitioning coefficient, aqueous solubility, brain/blood partition coefficient, Caco-2 cell permeability, serum protein binding, number of likely metabolic reactions, and others. Drug likeness (#stars), number of property descriptors from the full list of descriptors computed by the QikProp that fall outside the range of values determined for 95% of known drugs, was used as an additional compound selection filter.

## 4. Conclusions

In the current study, and as a continuation of our efforts to find new drug candidates for leukemia treatment, four new withanolide-type steroids, named withaperoxidins A–D, with an unusual cyclic peroxide moiety, and twenty-five known compounds were identified from *W. aristata*. Cyclic-endoporoxide natural products are very rare, and this is the first report of withasteroid-endoperoxides from *Withania* genus, suggesting that the enzymatic system in *W. aristata* is quite different to other *Withania* species. Biological evaluation and in silico ADME exploration revealed that four compounds from this series are promising anti-leukemic agents, showing higher effectiveness than the known chemotherapeutic drug etoposide. This potency is also coupled with a good selectivity index on non-tumoral cells and drug-likeness profile. SAR analysis provided new, valuable information on the pharmacophore for withanolide-type compounds, suggesting that chemical optimization of B, D, and lactone rings will be helpful in the rational design of anti-leukemic drugs. Therefore, the compounds identified deserve further studies to unravel their potential as a therapeutic alternative against leukemia. These results reinforce the potential of *Withania* species as a source of lead compounds and chemical diversity.

## Figures and Tables

**Table 1:** ^1^H-NMR spectroscopic data for compounds **1**–**4***^a^*.

H	1	2	3	4
2	4.53 d (6.2)	4.60 d (6.4)	4.53 d (6.5)	4.53 d (6.2)
3	6.69 dd (6.2, 8.2)	6.67 dd (6.4, 8.1)	6.70 dd (6.5, 8.4)	6.69 dd (6.2, 8.1)
4	7.04 d (8.2)	6.58 d (8.1)	7.04 d (8.4)	7.05 d (8.1)
6	4.08 s	4.15 s	4.08 s	4.10 s
7	1.70 α; 2.01 β, m	1.45 α; 2.01 β	1.62 α; 2.14 β, m	1.95 α; 2.14 β, m
8	1.88	1.98	1.83	2.01
9	1.90	1.03	1.92	1.99
11	1.61, 1.90	1.69, 2.26 m	1.60, 1.81	1.65, 1.70
12	1.50, 1.73	1.38, 1.69	1.23, 2.03	1.69, 1.73
14	1.75	1.59	1.19	1.55
15	1.25, 1.74	1.28, 1.72	1.20, 1.67	1.43, 2.11 m
16	1.63, 1.71	1.46, 2.00	1.40, 1.69	5.55 s
17			1.16	
18	0.88 s	0.88 s	0.79 s	0.88 s
19	1.31 s	1.61 s	1.31 s	1.35 s
20	2.35 m	2.35 m	2.04	2.54 m
21	1.06 d (7.0)	1.04 d (6.9)	1.05 d (6.7)	1.14 d (7.0)
22	4.69 t (8.2)	4.67 dt (2.9, 11.8)	4.46 dt (3.3, 13.3)	4.47 dt (3.9, 12.9)
23	2.56 d (8.0)	2.55 d (8.1)	2.02 α; 2.54 β, m	2.19 α, m; 2.56 β, m
27	4.37, 4.42 d_AB_ (12.6)	4.37, 4.41 d_AB_ (11.8)	4.38, 4.40 d_AB_ (12.2)	4.38, 4.42 d_AB_ (13.7)
28	2.05 s	2.04 s	2.08 s	2.06 s

*^a^* Spectra recorded in CDCl_3_ at 400 MHz. *J* in parenthesis in Hz. Signals without multiplicity assignments were overlapping resonances deduced by HSQC experiments.

**Table 2:** ^13^C-NMR spectroscopic data for compounds **1**–**4***^a^*.

C	1	2	3	4
1	206.0, C	205.9, C	206.0, C	206.2, C
2	78.6, CH	80.0, CH	78.6, CH	78.6, CH
3	126.5, CH	125.9, CH	126.4, CH	126.4, CH
4	141.4, CH	142.5, CH	141.3, CH	141.5, CH
5	84.2, C	84.6, C	84.1, C	84.3, C
6	67.1, CH	69.1, CH	67.1, CH	67.2, CH
7	36.6, CH_2_	36.4, CH_2_	31.1, CH_2_	31.2, CH_2_
8	29.6, CH	30.4, CH	29.4, CH	28.0, CH
9	42.1, CH	50.0, CH	42.4, CH	43.1, CH
10	48.2, C	48.8, C	48.2, C	48.5, C
11	22.0, CH_2_	23.1, CH_2_	22.2, CH_2_	22.2, CH_2_
12	32.0, CH_2_	31.9, CH_2_	39.4, CH_2_	34.8, CH_2_
13	48.3, C	48.3, C	42.9, C	47.2, C
14	49.3, CH	49.4, CH	54.8, CH	56.0, CH
15	23.6, CH_2_	23.6, CH_2_	24.3, CH_2_	34.3, CH_2_
16	34.9, CH_2_	34.0, CH_2_	27.2, CH_2_	124.4, CH
17	84.9, C	85.0, C	51.9, CH	155.3, C
18	15.0, CH_3_	15.1, CH_3_	12.0, CH_3_	16.5, CH_3_
19	19.4, CH_3_	16.0, CH_3_	19.5, CH_3_	19.5, CH_3_
20	42.7, CH	42.6, CH	38.8, CH	35.8, CH
21	9.4, CH_3_	9.4; CH_3_	13.4, CH_3_	16.9, CH_3_
22	79.2, CH	79.1, CH	78.8, CH	79.1, CH
23	32.9, CH_2_	32.9, CH_2_	29.8, CH_2_	32.9, CH_2_
24	154.3, C	154.1, C	152.9, C	152.6, C
25	125.1, C	125.2, C	125.6, C	125.7, C
26	167.1, C	167.1, C	166.7, C	166.4, C
27	57.4, CH_2_	57.5, CH_2_	57.4, CH_2_	57.5, CH_2_
28	19.9, CH_3_	20.0, CH_3_	19.9, CH_3_	19.9, CH_3_

*^a^* Spectra recorded in CDCl_3_ at 100 MHz. Data based on DEPT, HSQC, and HMBC experiments.

**Table 3:** Cytotoxic activity *^a^* (IC_50_, μM) of withanolides **1**–**29** against leukemia HL-60 and non-tumoral Vero cell lines.

Compound	HL 60	Vero	SI *^b^*
**1**	12.8 ± 0.24	>40	>3.1
**2**	26.7 ± 0.60	>40	>1.5
**3**	3.3 ± 0.45	16.0 ± 0.73	4.9
**4**	6.2 ± 0.10	31.6 ± 0.08	5.1
**5**	8.2 ± 0.09	6.4 ± 0.12	0.8
**6**	7.0 ± 0.55	11.5 ± 0.09	1.6
**7**	15.7 ± 0.04	>40	>2.6
**8**	4.2 ± 0.25	15.5 ± 0.35	3.7
**9**	14.8 ± 0.68	25.7 ± 0.65	1.7
**10**	16.8 ± 0.75	>40	>2.4
**11**	27.8 ± 0.45	>40	>1.4
**12**	6.6 ± 0.14	>40	>6.1
**13**	12.3 ± 0.11	23.8 ± 0.41	1.9
**14**	0.2 ± 0.02	6.4 ± 0.21	33.0
**15**	0.5 ± 0.07	4.6 ± 0.09	9.2
**16**	0.4 ± 0.06	5.7 ± 0.13	14.8
**17**	6.8 ± 0.30	38.6 ± 0.09	5.7
**18**	8.2 ± 0.43	34.7 ± 0.75	4.2
**19**	0.5 ± 0.05	1.9 ± 0.08	3.8
**20**	4.6 ± 0.35	10.0 ± 0.61	2.2
**21**	0.9 ± 0.05	6.2 ± 0.11	6.9
**22**	0.7 ± 0.09	16.9 ± 0.7	24.1
**23**	6.5 ± 0.55	>40	>6.2
**24**	>40	>40	1.0
**25**	1.5 ± 0.54	11.8 ± 0.1	7.9
**26**	13.4 ± 0.85	>40	>3.0
**27**	1.1 ± 0.2	7.8 ± 0.36	7.1
**28**	30.5 ± 0.45	>40	>1.3
**29**	19.2 ± 0.84	>40	>2.1
Etoposide *^c^*	2.4 ± 0.1	19.5 ± 0.16	8.2

*^a^* IC_50_: inhibitory concentration that reduces 50% of the cell viability. Values represent means ± standard deviation of three independent experiments, each performed in triplicate. *^b^* SI: selectivity index defined as Vero (IC_50_) on HL-60. *^c^* Etoposide was used as a positive control.

**Table 4:** In silico ADME profile prediction of selected withanolides *^a^* and their range/recommended values *^b^*.

Parameters	14	15	16	19	21	22	25	27	Range *^b^*
#stars	0	0	0	0	0	0	0	0	0–5
QPlogBB	−1.391	−0.825	−1.626	−1.358	−0.806	−1.397	−1.958	−1.63	−3.0 to 1.2
QPPCaco	226.10	615.13	188.31	242.52	634.31	189.69	94.33	128.79	<25 poor, >500 great
QPPMDCK	99.18	292.58	81.39	106.99	302.46	82.04	38.55	104.92	<25 poor, >500 great
QPlogKhsa	0.349	0.529	0.442	0.358	0.507	−0.1	0.204	0.711	−1.5 to 1.5
QPlogPo/w	3.054	3.55	3.341	3.123	3.537	2.501	2.456	3.833	−2.0 to 6.5
QPlogKp	−3.96	−3.302	−4.114	−3.824	−3.186	−4.204	−4.857	−4.355	−8.0 to −1.0
QPlogS	−5.138	−5.579	−6.077	−5.147	−5.495	−4.131	−5.438	−6.262	−6.5 to 0.5
#metab	4	4	4	6	6	3	6	5	1 to 8
%HOA	86.965	100	74.267	87.914	100	82.361	63.711	74.192	>80% high <25% poor
PSA	114.53	91.99	130.54	114.25	92.16	122.07	150.34	119.61	7.0 to 200.0
SASA	726.40	717.41	810.48	726.87	712.52	719.98	808.78	756.74	300.0 to 1000.0
Mol MW	470.61	454.61	512.64	468.59	452.59	468.59	530.66	507.07	130.0 to 725.0
#rotor	5	3	5	5	3	4	6	6	0 to 15
donorHB	1	1	1	1	1	0	2	2	0.0 to 6.0
accptHB	9.4	8.7	10.7	9.4	8.7	9.7	12.4	8.15	2.0 to 20.0
volume	1405.7	1386.1	1553.9	1409.1	1377.5	1393.9	1580.1	1459.5	500.0 to 2000.0

*^a^* Withanolides exhibiting IC_50_ values lower than those for etoposide on HL-60 cell line (IC_50_ < 2.4 μM). *^b^* Recommended values: #star (number of property values that fall outside the 95% range of similar values for known drugs), QPlogBB (predicted brain/blood partition coefficient), QPPCaco (predicted human epithelial colorectal adenocarcinoma cell line permeability in nm/s), QPPMDCK (predicted Madin–Darby canine kidney permeability in nm/s), QPlogKhsa (predicted binding to human serum albumin), QPlogPo/w (predicted octanol/water partition coefficient), QPlogKp (skin permeability), QPlogS (predicted aqueous solubility), #metab (number of likely metabolic reactions), % HOA (predicted human oral absorption on 0 to 100%), PSA (van der Waals surface area of polar nitrogen and oxygen atoms and carbonyl atoms), SASA (total solvent accessible surface area), MW (molecular weight), #rotor (number of non-trivial, non-hindered rotable bonds), donorHB (number of hydrogen-bond donor), accptHB (number of hydrogen-bond acceptor).

## Supplementary Materials

The following are available online. Figures S1–S7: NMR spectra of compounds **1**–**4**, Table S1: In silico ADME profile prediction of isolated withanolides **1**–**29**.
