# Supplementary material for: Withanolide-Type Steroids from Withania aristata as Potential Anti-Leukemic Agents
Source: Molecules. 2020 Dec 5;25(23):5744. doi: 10.3390/molecules25235744 (PMC7731379; doi:10.3390/molecules25235744)
Supplement: Supplementary file 1 [file molecules-25-05744-s001.pdf]

# Electronic Supporting Information

# Withanolide-type Steroids from *Withania aristata* as Potential Anti-leukemic Agents

**Laila M. Moujir [1], Gabriel G. Llanos[2], Liliana Araujo[1,3], Angel Amesty[2], Isabel L. Bazzocchi[2], and Ignacio A. Jiménez [2,*]**

1 Departamento de Bioquímica, Microbiología, Biología Celular y Genética. Facultad de Farmacia, Universidad de La Laguna, Avenida Astrofisico Francisco Sánchez s/n, 38206 La Laguna, Tenerife, Spain

2 Instituto Universitario de Bio-Orgánica Antonio González and Departamento de Química Orgánica, Universidad de La Laguna, Avenida Astrofísico Francisco Sánchez 2, 38206 La Laguna, Tenerife, Spain

*3* Carrera de Laboratorio Clínico. Facultad de Ciencias de la Salud, Universidad Nacional de Chimborazo, Avenida Antonio José de Sucre, Riobamba, Ecuador.

**Contents**

Figure S1 $^{1}$H and $^{13}$C NMR spectra for compound **1**

Figure S2 HMBC experiment for compound **1**

Figure S3 $^{1}$H NMR and $^{13}$C NMR spectra for compound **1a**

Figure S4 $^{1}$H NMR and $^{13}$C NMR spectra for compound **2**

Figure S5 HMBC spectra for compound **2**

Figure S6 $^{1}$H and $^{13}$C NMR spectra for compound **3**

Figure S7 $^{1}$H and $^{13}$C NMR spectra for compound **4**

Table S1 *In silico* ADME profile prediction of isolated withanolides **1**-**29**.

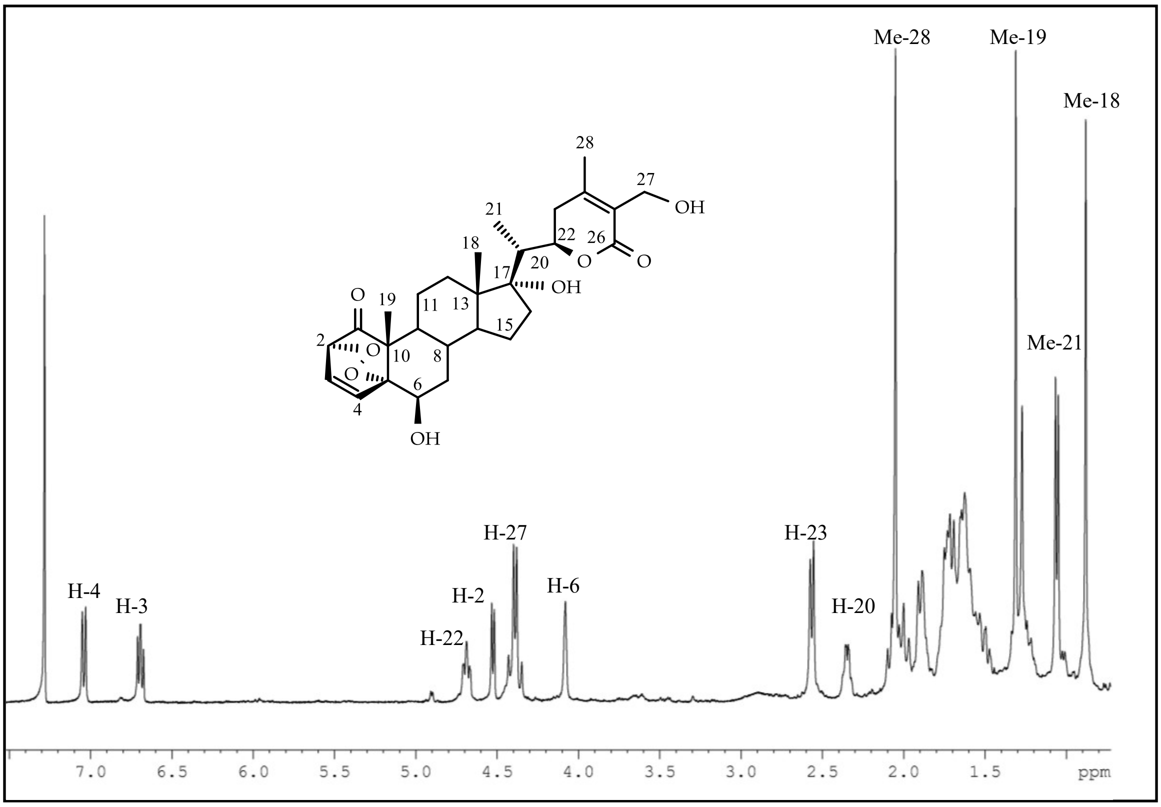


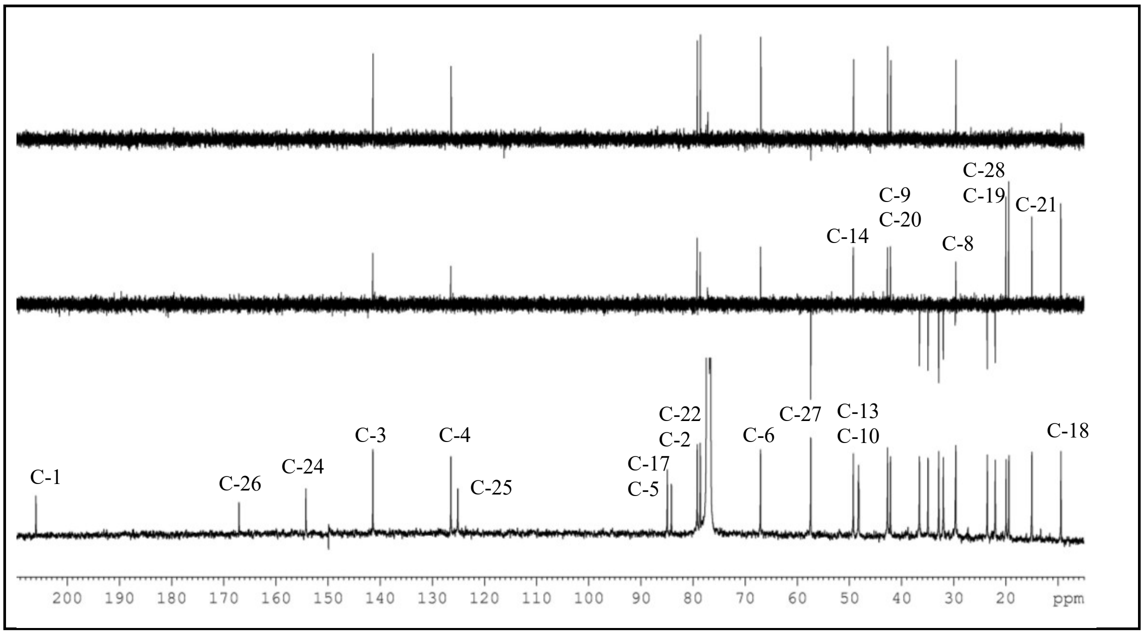


**Figure S1.** $^{1}$H NMR (400 MHz, solvent $CDCl_3$), $^{13}$C NMR spectrum and DEPT 90º and 135º experiments (100 MHz, solvent $CDCl_3$) of compound **1**.

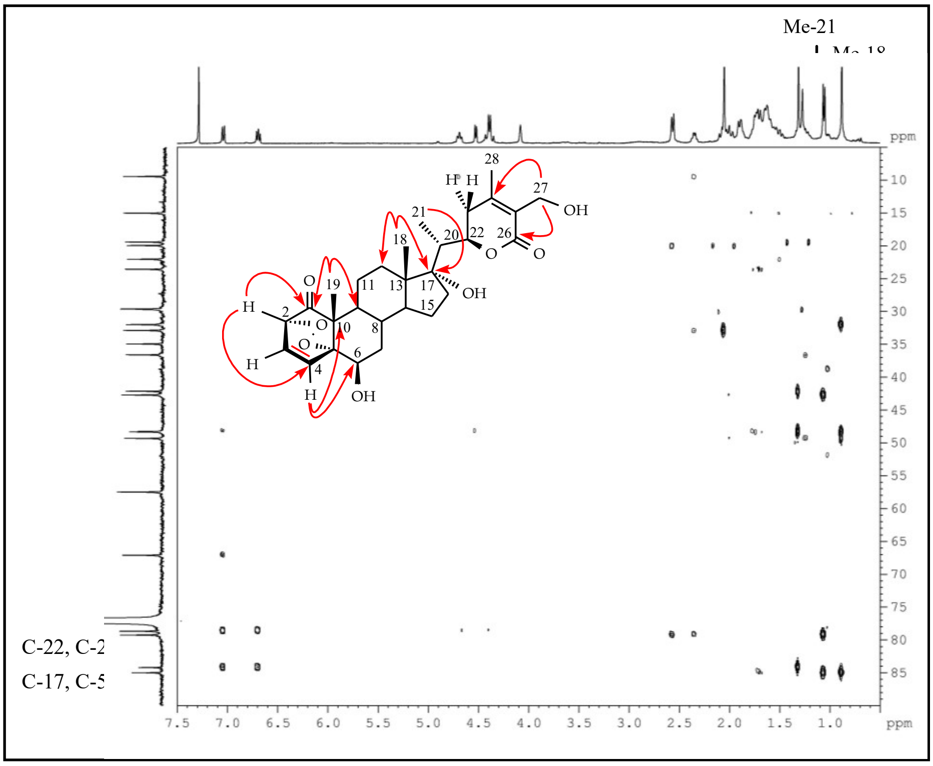


**Figure S2.** HMBC ($^{1}$H-$^{13}$C long-range) experiment (400 MHz, solvent $CDCl_3$) of compound **1**.

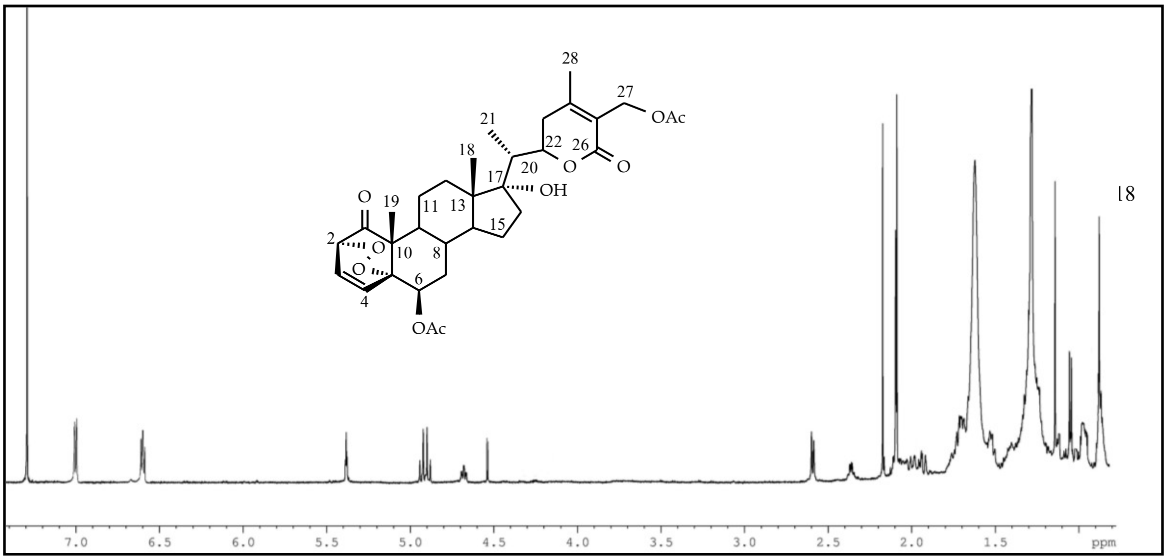


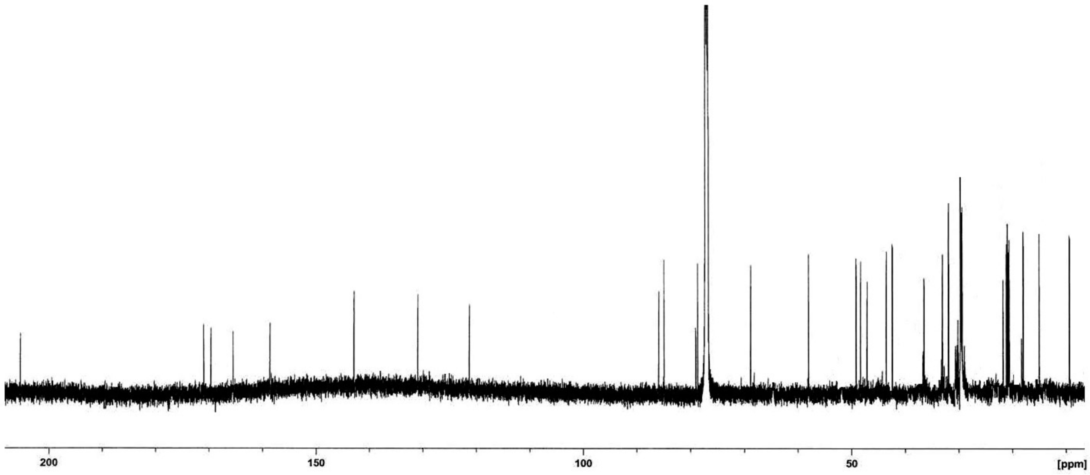


**Figure S3**. $^{1}$H NMR (400 MHz, solvent $CDCl_3$) and $^{13}$C NMR spectra (100 MHz, solvent $CDCl_3$) of derivative **1a**.

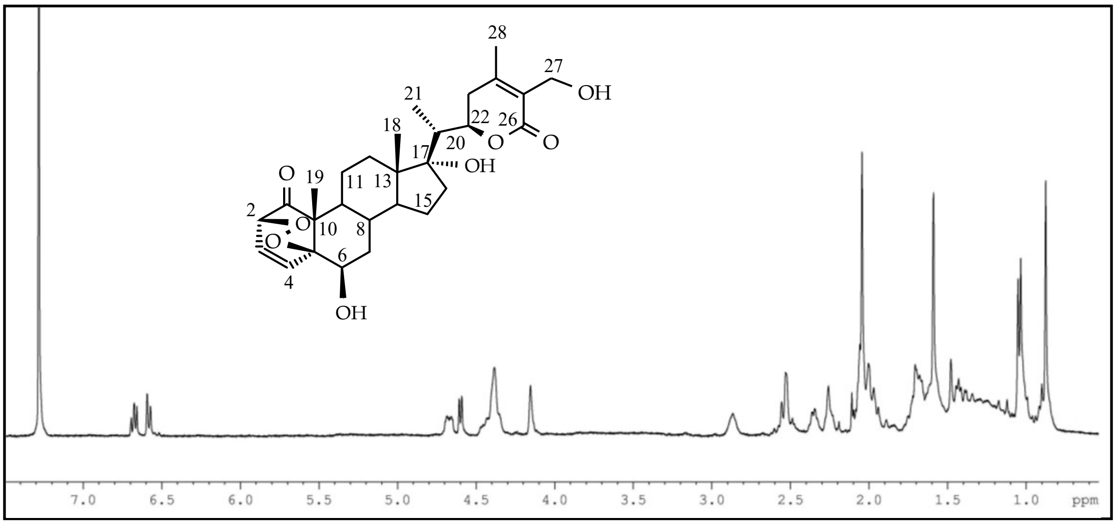


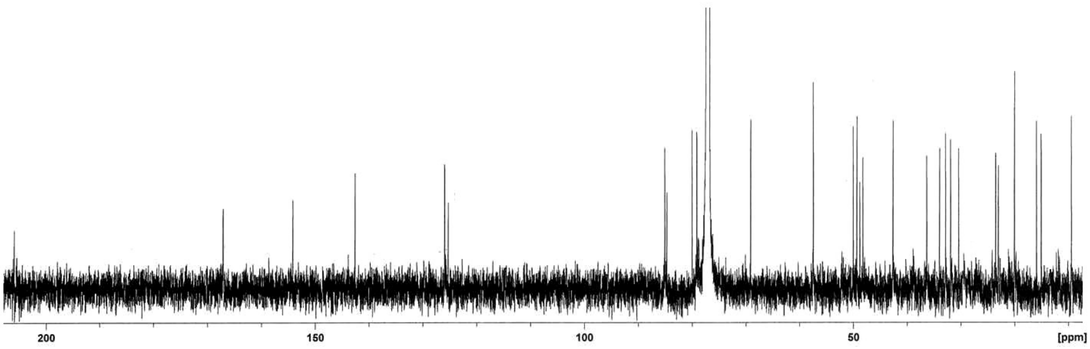


**Figure S4**. $^{1}H$ NMR (400 MHz, solvent $CDCl_3$) and $^{13}C$ NMR spectra (100 MHz, solvent $CDCl_3$) of compound **2**.

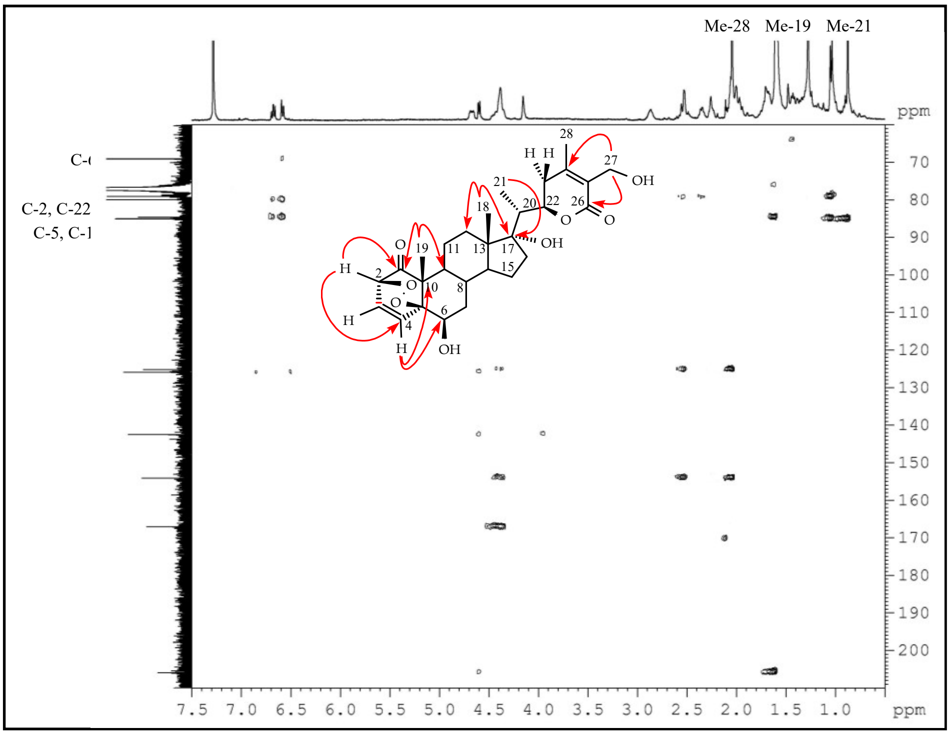


**Figure S5.** HMBC ($^{1}H$-$^{13}C$ long-range) experiment (400 MHz, solvent $CDCl_3$) of compound **2**.

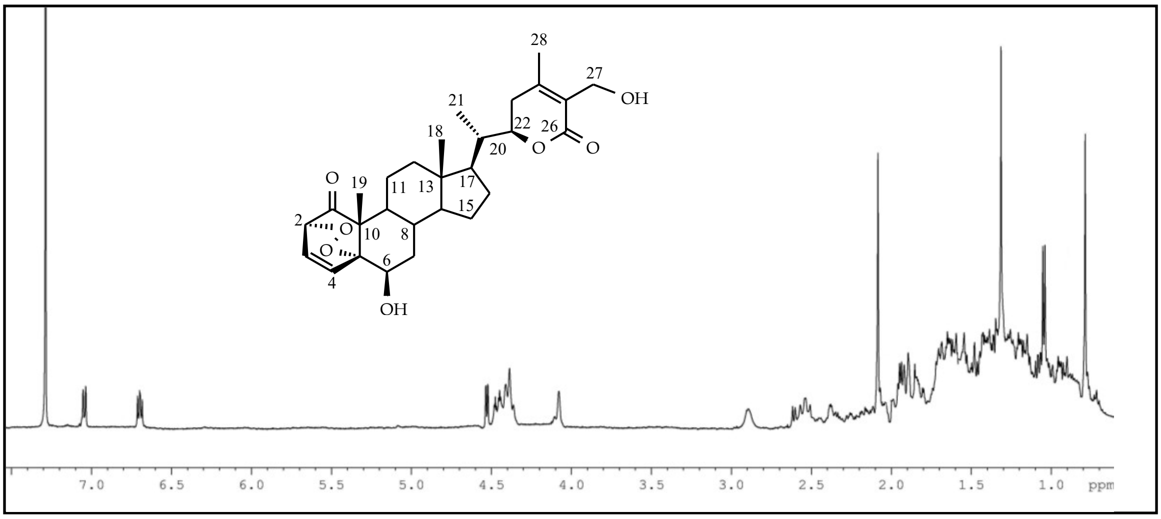


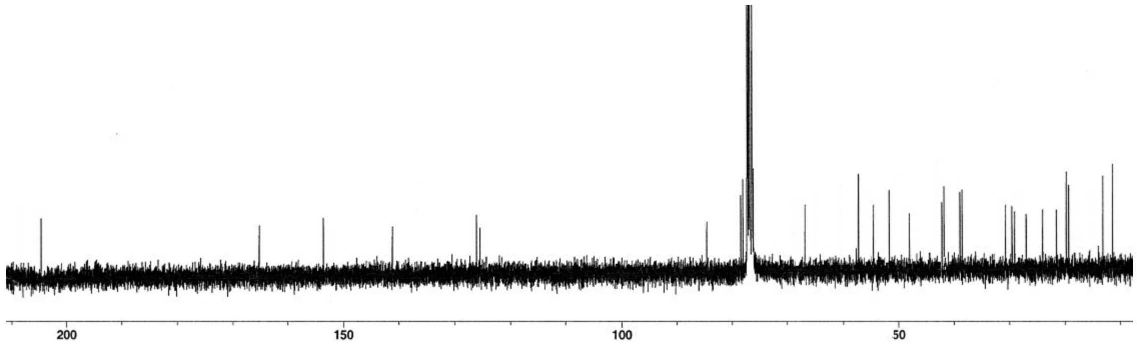


**Figure S6.** $^{1}H$ NMR (400 MHz, solvent $CDCl_3$) and $^{13}C$ NMR spectra (100 MHz, solvent $CDCl_3$) of compound **3**.

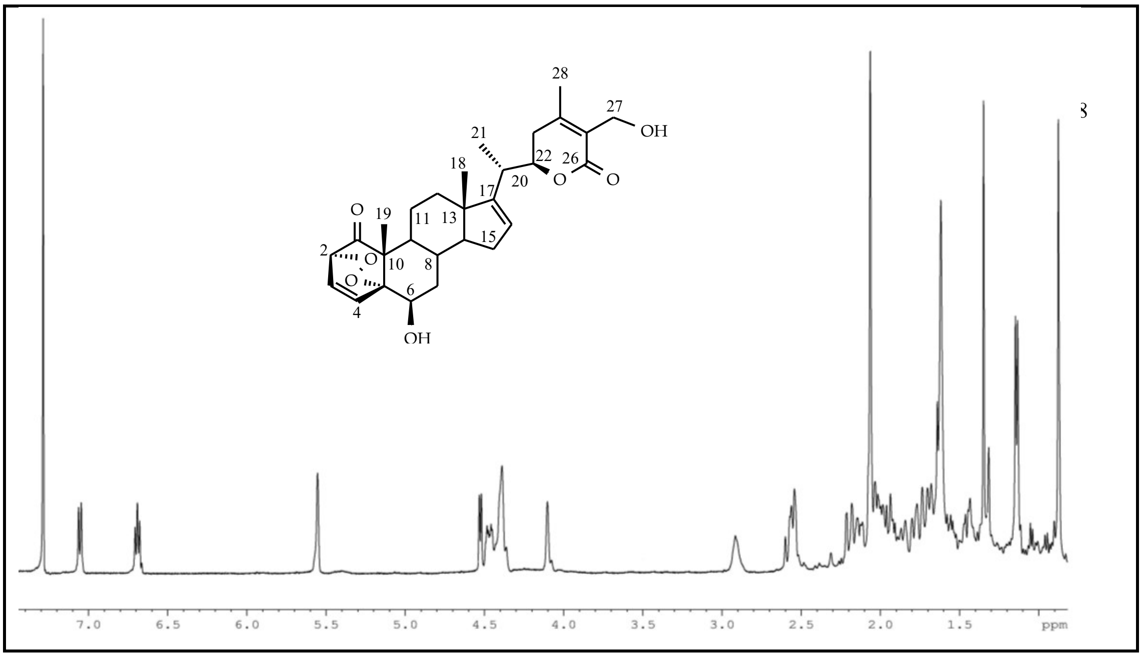

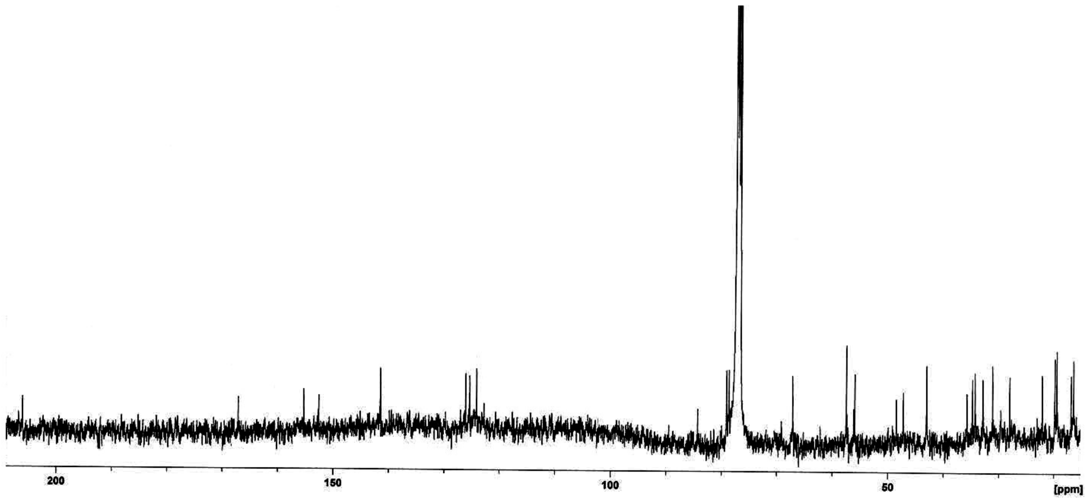

**Figure S7.** $^{1}H$ NMR (400 MHz, solvent $CDCl_3$) and $^{13}C$ NMR spectra (100 MHz, solvent $CDCl_3$) of compound **4**.

**Table S1**. Computational pharmacokinetic parameters (ADME) of isolated withanolides (**1-29**) from *Withania aristata*.

| compd | #stars | QPlogBB | QPPCaco | QPPMDCK | QPlogKhsa | QPlogPo/w | QPlogKp | QPlogS | #metab | %HOA | PSA | SASA | mol MW | #rotor | donorHB | accptHB | volume |
|---|---|---|---|---|---|---|---|---|---|---|---|---|---|---|---|---|---|
| **1** | 0 | -1,915 | 85,569 | 34,698 | 0,206 | 2,384 | -4,708 | -5,006 | 6 | 62,531 | 137,7 | 744,3 | 502,603 | 6 | 2 | 10,6 | 1438,1 |
| **2** | 0 | -1,951 | 78,919 | 31,793 | 0,204 | 2,35 | -4,784 | -4,999 | 6 | 61,7 | 138,2 | 743,9 | 502,603 | 6 | 2 | 10,6 | 1437,0 |
| **3** | 0 | -1,705 | 123,397 | 51,542 | 0,304 | 2,788 | -4,496 | -5,248 | 5 | 80,702 | 123,6 | 742,9 | 486,604 | 5 | 1 | 9,85 | 1427,2 |
| **4** | 0 | -1,67 | 132,677 | 55,744 | 0,313 | 2,857 | -4,359 | -5,259 | 7 | 81,666 | 123,3 | 743,6 | 484,588 | 5 | 1 | 9,85 | 1430,3 |
| **5** | 0 | -1,347 | 254,537 | 112,729 | 0,73 | 3,924 | -3,681 | -5,864 | 7 | 92,977 | 102,1 | 723,7 | 452,589 | 5 | 1 | 7,4 | 1393,3 |
| **6** | 0 | -0,786 | 681,937 | 327,075 | 0,903 | 4,4 | -3,041 | -6,29 | 7 | 100 | 79,6 | 714,1 | 436,59 | 3 | 1 | 6,7 | 1371,9 |
| **7** | 0 | -1,453 | 215,708 | 94,262 | 0,764 | 3,932 | -3,899 | -6,079 | 5 | 91,739 | 103,7 | 736,0 | 454,605 | 5 | 1 | 7,4 | 1407,8 |
| **8** | 0 | -1,442 | 194,313 | 84,199 | 0,45 | 3,576 | -4,007 | -5,435 | 7 | 88,841 | 110,9 | 733,5 | 450,574 | 4 | 0 | 7,7 | 1399,2 |
| **9** | 0 | -1,841 | 79,341 | 31,977 | 0,391 | 2,765 | -4,84 | -5,47 | 8 | 77,133 | 132,1 | 736,2 | 466,573 | 4 | 1 | 9,4 | 1408,4 |
| **10** | 1 | -1,446 | 239,335 | 105,47 | 0,876 | 4,151 | -3,883 | -6,813 | 8 | 93,828 | 104,3 | 768,3 | 452,589 | 4 | 1 | 7,4 | 1437,9 |
| **11** | 0 | -1,841 | 79,346 | 31,979 | 0,391 | 2,765 | -4,84 | -5,47 | 8 | 77,133 | 132,1 | 736,2 | 466,573 | 4 | 1 | 9,4 | 1408,4 |
| **12** | 0 | -1,828 | 104,283 | 42,969 | 0,436 | 2,974 | -4,426 | -5,401 | 6 | 80,48 | 124,8 | 738,0 | 470,605 | 6 | 2 | 9,1 | 1419,4 |
| **13** | 0 | -1,799 | 109,726 | 45,398 | 0,434 | 2,986 | -4,374 | -5,375 | 6 | 80,949 | 124,3 | 736,4 | 470,605 | 6 | 2 | 9,1 | 1418,1 |
| **14** | 0 | -1,391 | 226,096 | 99,179 | 0,349 | 3,054 | -3,96 | -5,138 | 4 | 86,965 | 114,5 | 726,4 | 470,605 | 5 | 1 | 9,4 | 1405,7 |
| **15** | 0 | -0,825 | 615,133 | 292,584 | 0,529 | 3,55 | -3,302 | -5,579 | 4 | 100 | 92,0 | 717,4 | 454,605 | 3 | 1 | 8,7 | 1386,1 |
| **16** | 0 | -1,626 | 188,305 | 81,388 | 0,442 | 3,341 | -4,114 | -6,077 | 4 | 74,267 | 130,5 | 810,5 | 512,642 | 5 | 1 | 10,7 | 1553,9 |
| **17** | 0 | -1,715 | 203,745 | 88,625 | 0,487 | 3,728 | -3,861 | -6,128 | 4 | 77,141 | 138,2 | 849,6 | 556,695 | 7 | 1 | 11,45 | 1663,7 |
| **18** | 0 | -1,737 | 102,755 | 42,289 | 0,112 | 2,127 | -4,623 | -4,775 | 7 | 75,408 | 135,4 | 730,3 | 484,588 | 5 | 2 | 11,1 | 1416,6 |
| **19** | 0 | -1,358 | 242,523 | 106,989 | 0,358 | 3,123 | -3,824 | -5,147 | 6 | 87,914 | 114,2 | 726,9 | 468,589 | 5 | 1 | 9,4 | 1409,1 |
| **20** | 0 | -0,732 | 717,391 | 345,493 | 0,49 | 3,523 | -3,099 | -5,336 | 6 | 100 | 90,6 | 703,3 | 452,589 | 3 | 1 | 8,7 | 1369,2 |
| **21** | 0 | -0,806 | 634,309 | 302,455 | 0,507 | 3,537 | -3,186 | -5,495 | 6 | 100 | 92,2 | 712,5 | 452,589 | 3 | 1 | 8,7 | 1377,5 |
| **22** | 0 | -1,397 | 189,69 | 82,035 | -0,1 | 2,501 | -4,204 | -4,131 | 3 | 82,361 | 122,1 | 720,0 | 468,589 | 4 | 0 | 9,7 | 1393,9 |
| **23** | 0 | -1,598 | 156,377 | 66,58 | 0,247 | 2,644 | -4,173 | -4,874 | 5 | 81,697 | 128,7 | 727,1 | 486,604 | 6 | 2 | 10,15 | 1416,4 |
| **24** | 0 | -1,63 | 137,035 | 57,725 | 0,129 | 2,266 | -4,541 | -4,592 | 6 | 78,46 | 132,3 | 729,0 | 488,62 | 6 | 2 | 11,1 | 1436,7 |
| **25** | 0 | -1,958 | 94,325 | 38,552 | 0,204 | 2,456 | -4,857 | -5,438 | 6 | 63,711 | 150,3 | 808,8 | 530,657 | 6 | 2 | 12,4 | 1580,0 |
| **26** | 0 | -2,192 | 53,958 | 21,079 | 0,304 | 2,441 | -4,993 | -5,152 | 6 | 72,236 | 141,1 | 746,9 | 488,62 | 7 | 3 | 9,85 | 1440,3 |
| **27** | 0 | -1,63 | 128,792 | 104,919 | 0,711 | 3,833 | -4,355 | -6,262 | 5 | 74,192 | 119,6 | 756,7 | 507,065 | 6 | 2 | 8,15 | 1459,5 |
| **28** | 0 | -2,007 | 68,422 | 27,248 | 0,245 | 2,986 | -4,901 | -4,819 | 4 | 77,277 | 134,4 | 737,6 | 470,605 | 6 | 0 | 8,4 | 1422,2 |
| **29** | 0 | -1,946 | 80,051 | 32,287 | 0,285 | 3,05 | -4,997 | -4,935 | 5 | 78,87 | 133,3 | 743,7 | 472,62 | 6 | 0 | 8,4 | 1436,0 |
| Range[a] | 0 to 5 | -3.0 to 1.2 | < 25 poor >500 great | < 25 poor >500 great | -1.5 to 1.5 | -2 to 6.5 | -8.0 to -1.0 | -6.5 to 0.5 | 1.0 to 8.0 | < 25 % poor >80% great | 7.0 to 200.0 | 30.0 to 1,000 | 130.0 to 725.0 | 0 to 15.0 | 0 to 6.0 | 0 to 20.0 | 500 to 2000 |

#star (number of property values that fall outside the 95% range of similar values for known drugs), QPlogBB (predicted brain/blood partition coefficient), QPPCaco2 (predicted human epithelial colorectal adenocarcinoma cell lines permeability in nm/s), QPPMDCK (predicted Madin-Darby Canine Kidney permeability in nm/s), QPlogKhsa (prediction of binding to human serum albumin), QPlogPo/w (predicted octanol/water partition coefficient), QPlogKp (skin permeability), QPlogS (predicted aqueous solubility), #metab (number of likely metabolic reactions), %HOA (Percent Human Oral Absorption), PSA (Van der Waals surface area polar nitrogen and oxygen atoms and carbonyl atoms), SASA (total solvent accessible surface area), MW (molecular weight), number of non-trivial, non-hindered ratable bonds). [a] Recommended values.
